# Economic benefits of subcutaneous trastuzumab administration: A single institutional study from Karolinska University Hospital in Sweden

**DOI:** 10.1371/journal.pone.0211783

**Published:** 2019-02-04

**Authors:** Elham Hedayati, Lionel Fracheboud, Vaidyanathan Srikant, David Greber, Susanne Wallberg, Christina Linder Stragliotto

**Affiliations:** 1 Department of Oncology-Pathology, Karolinska Institutet, Stockholm, Sweden; 2 Breast Cancer Flow, Patient Area of Breast Cancer Sarcoma and Endocrine Tumors, Theme Cancer, Karolinska University Hospital, Stockholm, Sweden; 3 The Boston Consulting Group AG, Zurich, Switzerland; University of South Florida, UNITED STATES

## Abstract

**Introduction:**

Adjuvant trastuzumab is a standard of care in the treatment of Human Epidermal growth factor Receptor 2 (HER2) positive early breast cancer (eBC). Initially trastuzumab could only be administered intravenously (IV), however since 2013, a subcutaneous (SC) formulation with comparable efficacy and safety profile is available and preferred by patients. Trastuzumab SC does not require pharmacy preparation and has shorter administration time. The objective of this study was to estimate the economic efficiency of the SC formulation of trastuzumab by assessing the economic benefits of actual SC-driven process changes at one single Swedish healthcare institution.

**Methods:**

This study analyzes changes in trastuzumab administration practice after the SC formulation was introduced at the Karolinska University Hospital. Process changes were identified and introduced in order to capitalize on the inherent work efficiency benefits of the SC formulation. Actual hospital data for 2015 were used to quantitatively estimate the annual economic impact of the changes. It encompassed administrative (i.e. non-medical) data of 178 newly diagnosed HER2-positive eBC patients and a total of 2,769 SC administrations. Realized economic benefits were expressed in hours saved by nurses, direct monetary cost savings and potential infusion fee revenue that could be earned through infrastructural revenue gains.

**Results:**

In 2015, the replacement of IV infusion to SC administration generated total time savings of more than 1,100 hours, and led to direct monetary cost savings of 603,000 EUR. It unlocked a capacity gain of 1–2 additional administrations daily within the existing facility infrastructure. Given the current remuneration structure per administration, this revenue gain translated into an incremental revenue potential of up to 3 million EUR.

**Conclusion:**

Data from this study showed that the shift from trastuzumab IV to SC formulation resulted in significant economic effects in terms of departmental resources related to time, direct monetary cost savings, and infrastructural revenue gains.

## Introduction

In Sweden, breast cancer (BC) is the most common cancer among women, and is responsible for one-third of all female cancer cases. In Stockholm County alone, approximately 1,700 women are diagnosed with early BC (eBC) annually, of which approximately 15% will overexpress the Human Epidermal growth factor Receptor (HER2) [1].

The HER2 positivity is associated with higher tumor aggressiveness and metastasis risk, and has therefore become an important target in the development of targeted therapies [2–4]. Trastuzumab (Herceptin) is one such therapy that binds to the HER2 receptor on the tumor surface and prevents tumor cell proliferation. It has become the standard of care in HER2-positive eBC and metastatic BC and has increased survival [5,6].

From its first European Union regulatory approval in the year 2000, trastuzumab has been administered intravenously (IV). However, since September 2013 a subcutaneous (SC) formulation of trastuzumab (Herceptin SC) is available which enables the delivery of trastuzumab via hand-held syringe or single-use injection device, thus obviating the need for intravenous infusion [7]. By way of direct comparison, a trastuzumab IV infusion requires 90-minute loading followed, if well tolerated, by subsequent doses over 30 minutes; while a 600 mg fixed SC dose of trastuzumab, is administered by manual injection/hand-held syringe in less than 10 minutes [7,8].

There is no significant difference concerning the severe adverse events defined according to the National Cancer Institute Common Terminology Criteria for Adverse Events (NCI CTCAE grade ≥3) version 3.0 between the two formulations [8]. Importantly, controlled clinical studies have demonstrated that trastuzumab SC is an effective treatment that is as well tolerated as trastuzumab IV, and preferred by patients most commonly because of the time saving as well as the reduction in pain and discomfort typically associated with IV infusions [8,9]. In addition to patient convenience benefits, the SC formulation has also been claimed to unlock economic efficiency gains for hospitals–and hence the health system as such–as a result of health care professional time savings and drug wastage reduction [10–12].

The objective of this study was to quantify the actual realized economic benefits associated with SC trastuzumab usage compared to trastuzumab IV. We analyzed trastuzumab SC usage within the Department of Oncology at the Karolinska University Hospital, which during 2015 handled 2,769 SC administrations.

## Methods

This paper synthesizes a single institutional study designed to estimate the actual annual economic benefits associated with switching from IV infusion to SC administration of trastuzumab for the department. The study was developed in two steps: first, a series of interviews with treating physicians and nurses was used to identify SC-induced changes in trastuzumab practice along the patient pathway (e.g. patient scheduling) and; second, realized benefits were quantified where possible on the basis of cost and revenue information for the calendar year 2015, obtained from the hospital's financial controlling department.

Dosing of trastuzumab IV is weight-based (8 mg/kg loading dose, 6 mg/kg maintenance doses every three weeks). The cost of trastuzumab IV is 609.92 EUR per vial containing 150 mg of trastuzumab and the cost of 600 mg fixed SC dose of trastuzumab is 1,792.39 EUR per administration [13]. We are not able to calculate the drug cost of trastuzumab IV, since no patient data including the weight of the patients was accessed.

### Patients

In total, 212 patients were diagnosed with HER2-positive BC at the Department of Oncology, Karolinska University Hospital during 2015. Of those, 34 patients did not qualify for SC treatment due to comorbidity or for body mass index reasons. Trastuzumab can be administered either in combination with chemotherapy or as a monotherapy. Calculations on benefits in terms of time and cost savings were performed on 178 SC-treated patients and SC administrations.

The total number of SC administrations during 2015 was 2,769, (of those 2,441 were mono administrations), since not all of the newly diagnosed patients received 17 cycles of treatment during the given calendar year. Quantitative data was obtained from hospital accounting and management information systems. No patient data was accessed at any point.

The interviewer during 2 days, scheduled approximately one hour meetings with i) two treating physicians both senior consultant at the breast cancer clinic, ii) two head nurses of the ward and the breast cancer clinic, iii) the head of “drug committee” at the clinic of oncology, iv) pharmacist and vi) hospital economy controller. No written informed consent was obtained. There wasn’t a strict interview guide as it was explorative in nature. There were general questions that were explored

### Benefit calculations

The quantification of benefits realized during the 2015 calendar year was based on number of newly diagnosed patients, SC administration data, standard operating procedures for administrations of IV vs. SC such as costs of consumables and time spent on preparation at the pharmacy, and on the administration at the ward. Information was collected from qualitative interviews with nurses, pharmacists and hospital administrators.

For the assessment of time savings, all recorded times were rounded to the nearest minute and based on a comparison of standard operating procedures for IV and SC. The total number of working days in Sweden is 240 days per year.

Direct monetary cost savings were estimated through the comparison of total cost associated with a standard IV and the associated SC trastuzumab cost. Sources for consumable costs were hospital and pharmacy data.

Realized revenue gains were derived by assessing the impact of SC-enabled optimizations of the patient scheduling practice at the hospital; i.e., the number of additional administrations (independent of treatment) possible within the existing infrastructure as a result of the switch from IV to SC. Potential revenue gain was estimated by multiplying the incremental number of administrations by the average reimbursement the hospital obtains per administration.

## Results

### Time savings for nurses

The first session of chemotherapy with trastuzumab IV takes on average 90 minutes whereas the subsequent cycles (cycle 2–17) takes 30 minutes for IV infusions. In contrast, SC trastuzumab takes 10 minutes to administrate, with no pharmacy preparation time. Given a total of 2,769 SC administrations for 178 newly diagnosed patients, the time savings corresponded to 1,101 hours which corresponded to 4.6 hours per working day of saved nurse time that is equivalent to 0.5 of a full-time nurse per year.

### Direct monetary cost savings

Comparison of costs per IV and SC administration showed that the use of SC allowed the hospital to save 603,000 EUR on direct monetary costs. This consisted of a) savings from avoided surgeries to implant port-a-caths (419,000 EUR), b) avoided fees the hospital has to pay the pharmacy for the reconstitution of IV doses (167,000 EUR), and c) the use of less expensive consumables in a SC setting (17,000 EUR). The cost for nurses’ time is not included since there is no time difference for nurses’ preparation of IV vs. SC administration. Underlying calculations are laid out in Table 1.

**Table 1:** Direct monetary cost savings.

**a) Direct monetary costs associated with avoided port-a-cath surgeries**	
Number of newly diagnosed EBC patients (i.e. do not receive port-a-caths)	178
Cost per port-a-cath implantation used for both chemo and IV administrations (EUR)	2,782
Cost per PICC line used for joint chemo administrations in SC setting (EUR)	428
Incremental cost savings per newly diagnosed patient (EUR)	2,354
** Total direct monetary cost savings from avoided surgeries to implant port-a-caths (EUR)**	**419,012**
**b) Direct monetary costs associated with pharmacy**	
Total number of SC administrations	2,769
Cost per IV reconstitution order at pharmacy (EUR)	60
Savings on regular reconstitution fees (EUR)	167,087
** Total direct monetary cost savings on pharmacy fees for the reconstitution of IV (EUR)**	**167,087**
**c) Direct monetary costs associated with materials for reconstitution and administrations**	
Total number of SC administrations	2,769
Material cost per IV administration at ward (EUR)	7.0
Cost of infusion needle (EUR)	6.3
Cost of bandage (EUR)	0.3
Cost of 3-way-perfusor (EUR)	0.4
Material cost per SC reconstitution and administrations at ward (EUR)	0.72
Cost of loading needle (EUR)	0.03
Cost of injection needle (EUR)	0.64
Cost of syringe (EUR)	0.05
Incremental cost savings of IV versus SC per administration (EUR)	6.28
Total material cost for IV administration at ward (EUR)	19,383
Total material cost for SC administration at ward (EUR)	1,994
**Total direct monetary cost savings on materials for SC reconstitution and administrations (EUR)**	**17,389**
**Total direct monetary cost savings**	
a) Avoided port-a-cath surgeries (EUR)	419,012
b) Avoided fees for reconstitution at pharmacy (EUR)	167,087
c) Materials for reconstitution and administrations (EUR)	17,389
**Total direct monetary cost savings (EUR)**	603,488

Direct monetary cost savings in Euros (EUR) on materials, administration, patient preparation and pharmacy fees on subcutaneous (SC) trastuzumab (Herceptin) administration compared to intravenous (IV) trastuzumab (Herceptin) infusion during 2015.

- Patients receiving SC therapy did not receive a port-a-cath implantation, which are otherwise required for IV infusions. Estimated all-inclusive costs per implantation are 2,782 EUR and the cost of a PICC line that is used instead of the port-a-cath in an SC setting is estimated to be 428 EUR. Hence, the SC-driven saving per newly diagnosed patient is 2,354 EUR. Given the number of 178 newly diagnosed patients with HER2-positive eBC, incremental savings on port-a-cath implantations amounted to 419,000 EUR.
- While IV administrations are reconstituted at the hospital pharmacy, SC doses are prepared by nurses at the ward. Since the hospital pays the pharmacy a flat fee per reconstituted IV dose, every SC administration comes with an implicit saving in pharmacy fees. With a total of 2,769 SC administrations and a base flat fee per IV reconstitution of 60 EUR, the hospital saved 167,000 EUR in 2015.
- Finally, SC administrations require a different, less expensive set of consumables than IV (e.g., syringe) infusions. Given the consumable cost difference of 6.27 EUR per administration and the total number of SC administration (2,769), this led to total cost savings of 17,400 EUR.

In summary, the total direct monetary cost savings amounted to 603,000 EUR with the greatest proportion attributable to avoided port-a-cath surgeries (69%), then avoided fees for reconstitution at pharmacy (28%), and finally materials for reconstitution and administration (3%).

### Capacity gain

Before SC trastuzumab was introduced, single IV trastuzumab infusions were scheduled in course of the afternoon following other longer lasting systemic infusions early in the morning. The short duration of a SC administration allowed the hospital to schedule SC mono administrations at the end of the day. Looking at any individual infusion chair, this change in patient scheduling created a gap between the longer lasting morning administration and the single SC trastuzumab in the evening. This time gap on average was long enough to treat 1–2 additional patient(s) per day and implied a higher chair turnover.

Of the 2,769 total SC administrations, 328 were given together with other therapies–and hence the patient could not be scheduled at the end of the day. The remaining 2,441 administrations created capacity for 1–2 additional patients per day, depending upon the durations of these additional patients' administration procedure.

### Implied incremental revenue potential through capacity gain

**Fig 1:**
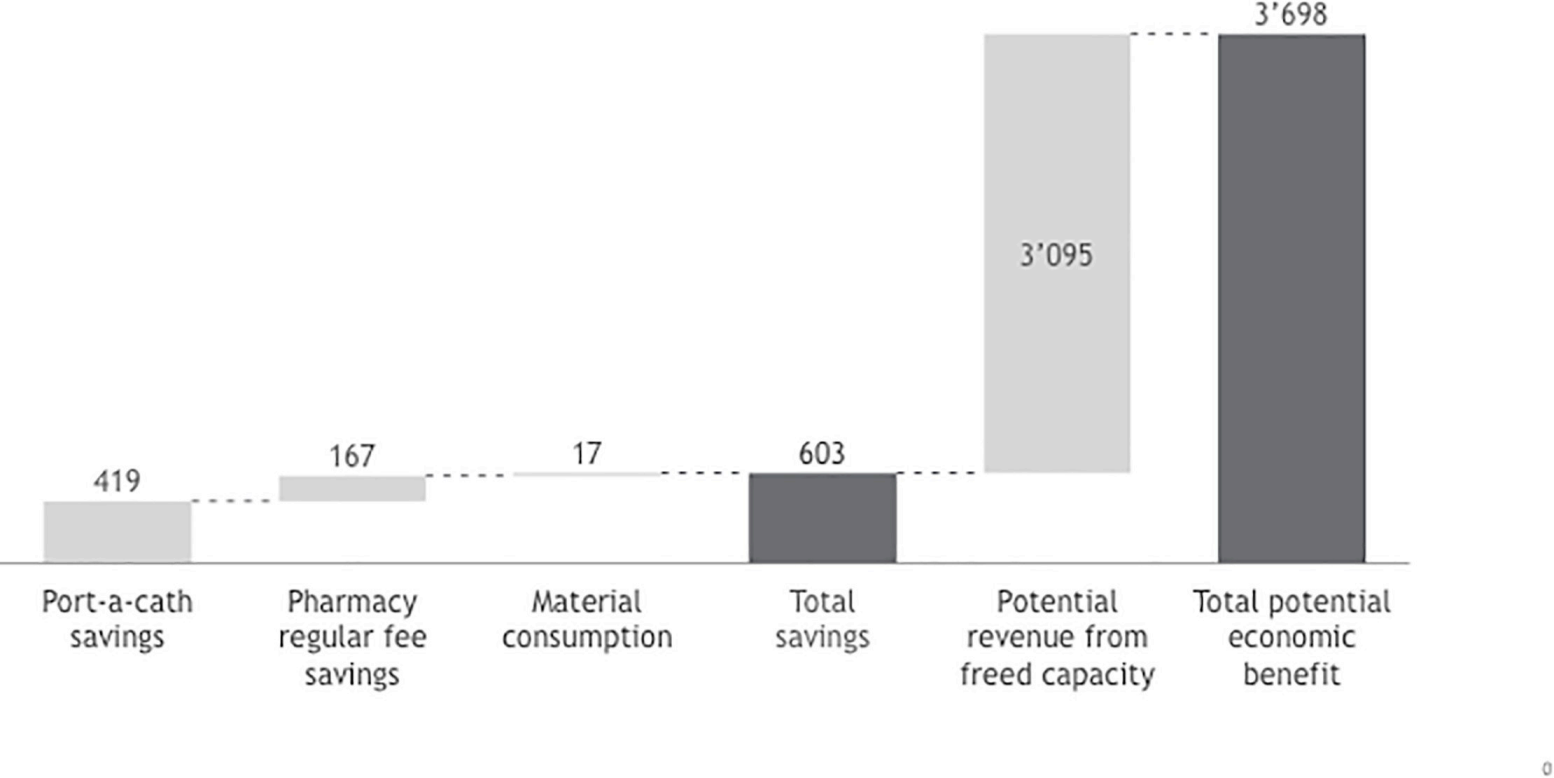
Summary of overall economic benefits encompassing cost savings as well as potential revenue gains through released capacity (thousands Euros; 2015).

From the perspective of the hospital, the ability to treat additional patients represents an incremental revenue potential of an estimated 1.5 million to 3.0 million EUR, depending on the patient mix. This calculation is based on the 2,441 SC mono administrations that create the potential for 1–2 additional patients per day to be treated, and average revenue per administration of 634 EUR (Fig 1).

## Discussion

This study of SC trastuzumab usage, compared to trastuzumab IV, documents quantifiable economic benefits of up to 3.6 million EUR in 2015 at the Karolinska University Hospital; of which 603,000 EUR represents actual direct monetary cost savings and the remainder corresponds to the maximum value of an implicit revenue gain. In addition, the hospital was able to save roughly 1,100 hours (4.6 per workday) on trastuzumab administration and nurse time, due to the shorter duration of an SC injection compared to an IV infusion. These time savings not only represent the time nurses can use to pursue other activities leading to an increased oncological day clinic capacity and shortening of the waiting list for other cancer patients, but also the time that patients treated with trastuzumab spent less at the hospital, contributing to their own quality of life.

In addition to direct monetary cost savings such as pharmacy fees and consumables, the introduction of SC unlocked capacity for additional administrations at the hospital without any additional infrastructural investment. In practice, this meant an SC-induced change in patient scheduling practice allowed the hospital to treat 1–2 additional patients for every trastuzumab mono administration given subcutaneously instead of intravenously. With the increase of the maximum daily chair capacity, the hospital managed to increase the total ward capacity and reduce the waiting list for all oncological day-care treatment without any infrastructural investment (e.g., additional chairs or rooms).

Costs for BC treatments are both resource-intensive and tend to increase due to the increasing number of diagnosed patients. This constitutes a major burden for the society. Any attempt to increase the efficiency of cancer care that aims to rationalize and reduce costs without compromising quality is a priority within healthcare. The purpose of this study was primarily to estimate economic benefits due to transition from IV infusion to SC administration of trastuzumab in a hospital setting. In the PrefHer study the patients and nurses preferred the SC administration above IV with reduced patient chair time and health care professional time [9]. Our results showed similarly a shorter patient chair time with SC administration through reduced nurse time. Today Sweden has a shortage of oncology nurses while the number of cancer patients is increasing and demands for oncological day-care treatments are rising. Our results showed 1,101 hours saved nurse time per year that is equivalent to 0.5 of a full-time nurse per year. This time saving was beneficial for both the oncological day clinic and the cancer patients since the waiting list for other cancer patients was reduced. Our results showed that SC administration resulted in a incremental revenue potential through time savings and financial savings, these results are similar to other studies analyzing SC formulation over IV infusions of trastuzumab [14,15].

Our analysis is based partially on interviews identifying SC-induced changes in trastuzumab practice along the patient pathway. It could be more precise with a prospective observational study such as other time and motion studies. However, the economic benefits due to practice changes were possible to be documented from the interviews and hospital's financial controlling department. Critically, indirect costs related to the time lost to receive treatment, including both time off work (production loss) and lost leisure time were not calculated in this study as the intent was to purely look at the financial impact from the perspective of the provider, namely Karolinska University Hospital.

In conclusion, this study tangibly demonstrates that the inherent efficiency benefits of the SC formulation over IV infusions can be translated into actual economic benefits for the providing center or hospital. It describes how one particular university hospital adapted its processes to cater to the specifics of the SC formulation and then logically deduces the causal impact of these process changes on hospital economics. Therefore, our study addresses the key shortfall of previous publications which tried to convert time savings into monetary gains without assessing if hospital operations had been adapted at all–the key criteria to realize economic benefits. These results are based on a single institutional study and should therefore be applied with caution in generalizing the results, particularly as every hospital operates in its own context. For the future, SC trastuzumab could be self-administered at home by the patient in order to improve the patients care including increase in patients satisfaction.

## Supporting information

S1 Table Explorative questions. (DOCX)

S1 File The dataset of cost and revenue information for the calendar year 2015, obtained from the hospital's financial controlling department. (XLSX)
