# Supplementary material for: Economic benefits of subcutaneous trastuzumab administration: A single institutional study from Karolinska University Hospital in Sweden
Source: PLoS One. 2019 Feb 4;14(2):e0211783. doi: 10.1371/journal.pone.0211783 (PMC6361452; doi:10.1371/journal.pone.0211783)
Supplement: S1 Table — (DOCX) [file pone.0211783.s001.docx]

| Explorative Questions |
| --- |
| **To treating physicians:** |
| - Do you treat all you patients with trastuzumab SC or are some still treated with trastuzumab IV? - Which patients are not appropriate to treat with trastuzumab SC? - Do you see any overall difference for your patients and/or yourself (regarding schedule and workload) since introduction of trastuzumab SC? |
| **To the nurses at ward:** |
| - What are the opening hours of the ward (Monday-Sunday)? - How many patients per chair per day do you have? - What is the daily chair capacity? - How many patients per bed per day? - What is the duration of trastuzumab IV and SC? - What is the duration of short (other than SC), medium lengths and long administrations? - Which material do you need for administration of trastuzumab SC vs. IV? |
| **To the pharmacist:** |
| - Trastuzumab IV is more at risk to be ordered late because patients do not have doctor visit every time --> doctor will order for the very treatment at the moment, but not further - How many acute/ late orders of trastuzumab IV? - What is the fee per IV reconstitution order at pharmacy? - What is the penalty fee for acute/ late order of trastuzumab IV? |
| **To the head of drug committee:** |
| - Total nr of administration of trastuzumab SC (2013, 2014, 2015)? - Total SC mono dose (mg)? - Dose per administration (mg)? - Total SC combination with chemotherapy dose (mg) - Total number of patients treated with trastuzumab SC |
| **To controller:** |
| - The revenue/visit at the ward? - Amount of nurses employed at the ward? - Total working days per year? - The working hours of the nurses? - Visits per day at the ward? - Amount of patients per treating nurse per day? - Total nr of beds at the ward? - Amount of patients/bed/day? - Number of port-a-cath patients? - The Cost of port-a-cath vs. PICC-line? - The equipment cost needed for administration of trastuzumab SC vs. IV? |
